# Mastering Your Fellowship: Part 1, 2023

**DOI:** 10.4102/safp.v65i1.5646

**Published:** 2023-01-05

**Authors:** Klaus B. Von Pressentin, Mergan Naidoo, Michéle Torlutter, Ts’epo Motsohi, Tasleem Ras

**Affiliations:** 1 Division of Family Medicine, Department of Family, Community and Emergency Care, Faculty of Health Sciences, University of Cape Town, Cape Town, South Africa; 2 Department of Family Medicine, University of KwaZulu-Natal, Durban, South Africa; 3 Department of Family Medicine and Primary Care, University of the Witwatersrand, Johannesburg, South Africa; 4 Division of Family Medicine and Primary Care, Department of Family and Emergency Medicine, Faculty of Medicine and Health Sciences, Stellenbosch University, Cape Town, South Africa

**Keywords:** Family physicians, FCFP (SA) examination, family medicine registrars, postgraduate training, national exit examination, ear, nose and throat conditions

## Abstract

The series ‘Mastering your Fellowship’ provides examples of the question formats encountered in the written and clinical examinations, Part A of the Fellowship of the College of Family Physicians of South Africa (FCFP [SA]) examination. The series is aimed at helping family medicine registrars (and their supervisors) in preparing for this examination.

This section in the *South African Family Practice* journal is aimed at helping registrars in preparing for the Fellowship of the College of Family Physicians (FCFP [SA]) Final Part A examination and will provide examples of the question formats encountered in the written examination: multiple-choice questions (MCQ) in the form of single best answer (SBA – Type A) and/or extended matching questions (EMQ – Type R); short answer questions (SAQ), questions based on the critical reading of a journal article (CRJ: evidence-based medicine) and an example of an objectively structured clinical examination (OSCE) question. Each of these question types is presented based on the College of Family Physicians blueprint and the key learning outcomes of the FCFP (SA) programme. The MCQs are based on the 10 clinical domains of family medicine, the SAQs are aligned with the five national unit standards and the critical reading section will include evidence-based medicine and primary care research methods.

This edition is based on unit standard 1 (effectively manage themselves, their team and their practice, in any sector, with visionary leadership and self-awareness, to ensure the provision of high-quality, evidence-based care), unit standard 2 (evaluate and manage patients with both undifferentiated and more specific problems cost-effectively according to the bio-psychosocial approach) and unit standard 5 (conduct all aspects of health care in an ethical and professional manner). The clinical domain covered in this edition is ear, nose and throat (ENT). We suggest that you attempt to answer the questions (by yourself or with peers or supervisors) before finding the model answers online: http://www.safpj.co.za/.

Please visit the Colleges of Medicine website for guidelines on the Fellowship examination: https://www.cmsa.co.za/view_exam.aspx?QualificationID=9.

We are keen to hear about how this series is assisting registrars and their supervisors in preparing for the FCFP (SA) examination. Please e-mail us (editor@safpj.co.za) with your feedback and suggestions.

## Multiple choice question (MCQ): Single best answer

A 70-year-old male patient presents with epistaxis to the emergency centre (EC). The patient is bleeding profusely, and the team cannot localise the source of the bleeding. The patient’s vital signs are as follows: blood pressure = 160/80 mmHg, pulse = 108 beats/min, respiratory rate = 24 breaths/min, temperature = 37.5 °C. He has no other evidence of bleeding. The patient has been pinching his nose for the last 10 minutes. The bleeding continues when the pressure is released. You note that the team on call is a community service medical officer and two interns. They phone you for advice at 23:00. What is the most appropriate next step?

a) Administer intravenous tranexamic acid.

b) Insert a compressed nasal sponge.

c) Insert a Foley catheter and inflate.

d) Lower the blood pressure.

e) Pack the anterior nasal cavity with gauze.

*Answer:* b)

Model answers

Epistaxis is a relatively common condition, although the actual incidence is unknown because most cases self-abort and are managed at home. Severe epistaxis requires prompt evaluation in the EC and appropriate resuscitation. A focused history noting the duration, severity of the haemorrhage and the side of initial bleeding. Enquire about previous epistaxis, hypertension, hepatic or another systemic disease, family history, easy bruising or prolonged bleeding after minor surgical procedures. Recurrent episodes of epistaxis, even if self-limited, should raise suspicion of significant nasal pathology. Use of medications, especially aspirin, nonsteroidal anti-inflammatory drugs, warfarin and heparin, should be documented, as these predispose to epistaxis. The examination using a light source is essential in establishing the point of bleeding. Applying vasoconstrictor drops may slow the bleeding, allowing for an accurate source assessment. Patients should be educated about first aid, which includes pinching the nose and applying an ice pack to the forehead while leaning forward.

The relationship between hypertension and epistaxis is not well understood. Patients with epistaxis commonly present with elevated blood pressure. Epistaxis is more common in hypertensive patients due to long-standing vascular fragility. Hypertension, however, is rarely a direct cause of epistaxis. More commonly, epistaxis and the associated anxiety cause an acute elevation of blood pressure. Therefore, therapy should focus on controlling haemorrhages and reducing anxiety as the primary means of blood pressure reduction.

Insert pledgets soaked with an anaesthetic-vasoconstrictor solution into the nasal cavity to anaesthetise and shrink nasal mucosa. Nasal packing is the usual practice in most settings in South Africa but is often poorly done and requires some skill. Packing is commonly performed incorrectly, using an insufficient amount of packing set primarily in the anterior naris. The gauze is a plug rather than a haemostatic pack when placed in this way. Physicians inexperienced in the proper gauze pack placement should use a nasal tampon or balloon instead.

A compressed sponge (e.g. Merocel^®^) is trimmed to fit snugly through the naris. Moisten the tip with surgical lubricant or topical antibiotic ointment. Firmly grasp the length of the sponge with bayonet forceps, spread the naris vertically with a nasal speculum and advance the sponge along the floor of the nasal cavity. Once wet with blood or a small amount of saline, the sponge expands to fill the nasal cavity and tamponade bleeding. The procedure requires very little skill and is suitable for all levels of emergency care doctors.

Another easy method of gaining control of bleeding in the anterior naris is nasal balloons, available in different lengths. A carboxymethyl cellulose outer layer promotes platelet aggregation. The balloons are as effective as nasal tampons, easier to insert and remove and more comfortable for the patient. To insert the balloon, soak its knit outer layer with water, insert it along the floor of the nasal cavity and inflate it slowly with air until the bleeding stops. These balloons are not readily available in most public sector hospitals in South Africa.

Further reading

- Naidoo M. Chapter 88: How to manage epistaxis. In: Mash B, et al, editors. South African Family Practice Manual. 4th ed. Braamfontein: Van Schaik; In press 2023.
- Traboulsi H, Alam E, Hadi U. Changing trends in the management of epistaxis. Int J Otolaryngol. 2015: 2015;263987. https://doi.org/10.1155/2015/263987.
- Bamimore O, Silverberg MA. Acute epistaxis [Internet]. 2022. New York: Medscape. [cited 2022 Sept 12]. Available from: https://emedicine.medscape.com/article/764719-overview.

## Short answer question (SAQ): The family physician’s role as a leader of clinical governance and capacity builder within the domain of ENT and antimicrobial stewardship

You are the family physician working in a community health centre. A medical officer (MO) working in the paediatric clinic alongside primary health care (PHC) nurses commented that she has recently seen a few children with hearing loss as a complication of otitis media (OM). At the same time, it is noted in the Pharmaceuticals and Therapeutics Committee (PTC) meeting that there is an increased need for antimicrobial stewardship in the management of common upper respiratory tract infections (URTIs).

1. As a leader of clinical governance in the clinic, what initial steps would you take to investigate this problem in the clinic? Describe three different approaches you might take. **(6 marks)**
2. Based on your findings, you decide to do a quality improvement project (QIP) on one of your findings. Describe the process you would follow. Apply a relevant example to this process in line with one of your responses to question 1. **(6 marks)**
3. You plan a continuing professional development (CPD) meeting to address the knowledge gap. List four important learning outcomes written in the correct format which address pertinent points in the management of OM in children. **(4 marks)**
4. Acquired antibiotic resistance and antimicrobial stewardship raise several ethical dilemmas regarding public health when it comes to balancing harms and benefits. Over a million deaths per year are attributable to resistant bacterial infections. Describe two ethical dilemmas relevant to primary care practice that you will broach in your CPD meeting to raise awareness. **(4 marks)**

**Total: 20 marks**

Model answers

### 1. As a leader of clinical governance in the clinic, what initial steps would you take to investigate this problem in the clinic? Describe three different approaches you might take. (6 marks)

(Provide any three approaches from the list below with a relevant example)

- File audit – Determine the current standard of care being provided and if this aligns with treatment guidelines. Also consider antibiotic stewardship, appropriate prescription of antibiotics, quality of note keeping and the number of children presenting with OM or URTI.
- Skills assessment and audit – Assess the competence of the staff who are new, and on an ongoing basis, assess the turnover of staff and provision of relevant supervision and training; note attendance at CPD meetings on the topic and observed consultations.
- Exploring problems in teams – apply root cause analysis methods, such as asking the 5 why’s, using the fishbone template and applying process mapping techniques. This may assist in understanding where breakdowns are occurring regarding health system factors or process issues, health care worker–related factors and patient factors. These may include problems with patient load, lack of access to functional equipment (otoscope), a gap in knowledge in treatment guidelines, poor examination technique and patient medication adherence.
- Explore learning needs and gaps – This may be on an individual level (doctors and PHC nurses), or it may be a priority and relevant for district health services and outcomes. Analyse and understand your intended audience and clarify their learning needs and gaps, which will in turn assist in developing learning objectives.
- Any other relevant response.

### 2. Based on your findings, you decide to do a quality improvement project (QIP) on one of your findings. Describe the process you would follow. Apply a relevant example to this process in line with one of your responses to question 1. (6 marks)

The current situation has been explored in question 2.1. The next steps will be to: *(Need to mention the step and elaborate with a relevant example for the mark)*

- Form a relevant team (including PTC committee members) – For example, family physician, MO and PHC nurse from paediatric clinic, pharmacist and facility manager.
- Agree on problem definition, criteria and set target standards – Apply to one of the above examples.
- Identify gaps in current provision – Apply to one of the above examples above.
- Analyse causes and explore ways to improve the situation – Apply to one of the above examples above.
- Planning and implementing the change – Apply to one of the above examples above.
- Sustain change – Apply to one of the above examples above. The cycle continues until the desired quality is achieved. The criteria used and the performance levels can be adjusted if necessary before the start of a new cycle (as per the principle of continuous quality improvement [QI]).

### 3. You plan a continuing professional development (CPD) meeting to address the knowledge gap. List four important learning outcomes written in the correct format which address pertinent points in the management of OM in children. (4 marks)

*Background information (not part of the model answer)*: In higher education today, teaching activities are not defined in terms of the content but rather in terms of the intended outcomes for the learners (see Bloom’s taxonomy). In other words, a learning outcome should specify what the learner should be able to do at the end of the teaching session. The learning outcome can be for knowledge, skills or attitudes, and the level of Bloom’s taxonomy should be clear from the verb used – list, describe, demonstrate.

At the end of your teaching activity, you should be able to:

- *Know or understand* (cognitive domain: knowledge or application of knowledge in problem-solving or critical reflection) – Possible knowledge learning outcomes may relate to indications, contraindications, anatomy, equipment, drugs, fluids and aftercare.
- *Be able to do* (psychomotor domain: skills) – Possible learning outcomes related to skill refer to performing the procedure.
- *Attitudes displayed* (affective domain: values and attitudes) – Possible learning outcomes related to attitude may relate to communication, caring and consent.

The content relating to the South African national guidelines for the management of upper respiratory tract infections should be expressed in the learning outcomes.

*The model answer should include any four options from the list below, preferably covering each domain: knowledge, skills and attitudes.*

- At the end of this session, you should be able to list the common organisms that cause OM.
- At the of this session, you should be able to discuss the primary preventative measures that have reduced the incidence of OM in children.
- At the end of this session, you should be able to demonstrate the correct examination of the ear using pneumatic otoscopy and tympanometry.
- At the end of this session, you should be able to list the diagnostic criteria for acute OM.
- At the end of this session, you should be able to describe an approach to rational antibiotic prescribing for acute OM.
- At the end of this session, you should be able to list conditions under which antibiotics should be prescribed for acute OM and when a more conservative approach can be taken.
- At the end of this session, you should be able to demonstrate how you counsel a carer or parent on when management with antibiotics may be required and on the issue of antibiotic adherence.

### 4. Acquired antibiotic resistance and antimicrobial stewardship raise several ethical dilemmas regarding public health when it comes to balancing harms and benefits. Over a million deaths per year are attributable to resistant bacterial infections. Describe two ethical dilemmas relevant to primary care practice that you will broach in your CPD meeting to raise awareness. (4 marks)

*The model answer should include any two well-described points for 2 marks each.*

- Primordial prevention and social determinants of health – Even when antibiotics are used scrupulously in individual patients, they can still acquire resistant organisms through no fault of their own from contact with infected or colonised people, animals and other environmental reservoirs. The medical fraternity should raise awareness and influence policy as a public health measure, including environmental and infection control policies.
- Distributive justice – Overuse of antibiotics in general practice may be because of a lack of evidence-based use by health practitioners, other incentives for health care workers or pressure from patients. Overuse in individuals may result in the depletion of a common resource for all. This requires regulation of human behaviour and may even require regulating access to a common resource for the greater good.
- Beneficence versus nonmaleficence – Antibiotic use is not a free ride; each use involves risk, and risk is more concentrated in the frequent user. Antibiotic consumption should require regulation. However, governance of antibiotic use through idealised prescription guidelines faces multiple real-world challenges – prescribers, agents and conflicts of interest. Clinicians may prioritise their immediate patients over the interest of other, distant or future patients. Antibiotics may also not be in the interest of the individual or the wider community.

Further reading

- Brink AJ, Cotton MF, Feldman C, et al. Updated recommendations for the management of upper respiratory tract infections in South Africa. S. Afr. Med. J. 2015;105(5):345–52.
- Moodley K. Chapter 10.8: Family medicine ethics - the four principles of medical ethics. In: Mash B, editor. Handbook of Family Medicine. 4th ed. Cape Town: Oxford University Press, 2017; p. 418–422.

## Critical appraisal of quantitative research

Read the accompanying article carefully and then answer the following questions. As far as possible use your own words. Do not copy out chunks from the article. Be guided by the allocation of marks concerning the length of your responses.

- Biagio L, Swanepoel DW, Laurent C, Lundberg T. Paediatric otitis media at a primary healthcare clinic in South Africa. S. Afr. Med. J. 2014;104(6):431–5.

**Total: 30 marks**

1. Did the study address a focused question? Discuss. (3 marks)
2. Identify three arguments the author made to justify and provide a rationale for the study. (3 marks)
3. Explain why a quantitative research methodology may be most appropriate for this research question. Comment on where and how a qualitative data collection methodology might still be applicable. (2 marks)
4. Critically appraise the sampling strategy. (5 marks)
5. Critically appraise how well the authors describe the data collection process. (5 marks)
6. Explain the difference between point prevalence and incidence. (2 marks)
7. Critically appraise the analysis and conclusions of the study. (4 marks)
8. Use a structured approach (e.g. relevance, education, applicability, discrimination, evaluation, reaction [READER]) to discuss the value of these findings to your practice. (6 marks)

Model answers

### 1. Did the study address a focused question? Discuss. (3 marks)

- The authors aimed to measure the prevalence of otitis media in a South African primary health care (PHC) clinic, Witkoppen Health and Welfare Centre.
- The question is focused as it describes the population of interest (paediatric population attending a PHC clinic) and the condition or phenomenon of interest (point prevalence of otitis media in this population) in a particular community or area (the Diepsloot community north of Johannesburg, South Africa).
- The authors wished to diagnose the condition of interest with greater sensitivity and specificity than either otoscopy or pneumatic otoscopy, by using otomicroscopy to diagnose and classify otitis media as a cause of middle-ear pathology in children.

### 2. Identify three arguments the author made to justify and provide a rationale for the study. (3 marks)

- Otitis media point prevalence in South Africa has never been measured, and most deaths from complications of otitis media are in sub-Saharan Africa and India. Chronic serous otitis media is also the most common cause of hearing impairment. This makes the study socially and scientifically relevant.
- Most studies of the prevalence of otitis media measure prevalence in children of school-going age and not in younger preschool children, who are more prone to otitis media.
- Early medical intervention is indicated in communities where chronic suppurative otitis media rates are more than 4%, as this constitutes a high-risk population. This supports the need to employ diagnostic methods to measure the point prevalence more accurately.

### 3. Explain why a quantitative research methodology may be most appropriate for this research question. Comment on where and how a qualitative data collection methodology might still be applicable. (2 marks)

- By definition, prevalence is a quantitative measure of proportion and depicts the proportion of a defined population with a disease or illness at a specified time. Therefore, measuring a proportion would require a quantitative methodology and is impossible to achieve using qualitative data and methods.
- Given that otomicroscopy was used for the first time in this setting, the study could conceivably be amended to address the additional objective of assessing the otologist’s experiences of otomicroscopy in primary health care. Perhaps the caregiver who brought the children would be interviewed for qualitative data on their experience of the process.

### 4. Critically appraise the sampling strategy. (5 marks)

- The researchers selected a specific primary healthcare clinic for their study. The clinic is a specialist care centre for primary health care paediatric human immunodeficiency syndrome (HIV) and tuberculosis (TB) patients. This also already indicates that it does not represent the more typical primary health care clinics in the country, which serve patients with all forms of illness. The more accurate description in the title of this study should be that of measuring the prevalence of OM in an HIV and TB primary healthcare clinic.
- Furthermore, the sampling was not random but consecutive. They recruited 140 children aged 2–16 years as a sample from registered clinic patients known to the service: the participants were recruited from the entire paediatric population attending the clinic for any purpose, whether for a routine clinic appointment or for chronic or acute treatment.
- They do not indicate on which days they consecutively collected samples and whether they sampled equally for each day of the week. They only specified that the on-site data collection continued over the course of 2 weeks.
- Bias could be introduced in this way of sampling if, for example, a specific type of child (age or illness) tends to come to the clinic on some days more than others.
- The researchers do not indicate how they calculated the sample size. This always affects the precision of the estimate of prevalence. Often, it is helpful to use prevalence rates from the literature to calculate sample size estimates.

### 5. Critically appraise how well the authors describe the data collection process. (5 marks)

- The authors described the collection of demographic data under the study population subheading in the methods section and not under the data collection subheading. It would have made more sense to include this data collection step in the data collection subsection, as this information was included in the data set.
- The authors did not specify who collected this information, and it seems like this information might have been captured by a research assistant or the specialist otologist, linked to the informed consent process and possibly the otomicroscopy assessment. It is important to note the person(s) who collected the data from the patients and parents or caregivers as well as the background of the data collectors.
- It was not clear whether the clinical notes and medical history from the patient’s folder were consulted to complement the dataset and verify the accuracy of comorbid risk factors described in the introduction section (host-related and environmental factors). It would have been useful to present the demographic and medical background data collection instrument as a supplement. Interestingly, even though this clinic served as a specialist HIV and TB centre, the researchers were not able to collect clinical data on HIV status. They mentioned that ‘ethical clearance did not allow for this’ but do not specify the reasons behind this (whether it was a protocol design flaw or whether this was a specification from the ethics review board).
- The data collection subsection in the methods section describes the technical process of otomicroscopy, including the type of device used (a Leica M525 F40 surgical microscope). The key elements captured by the specialist otologist are described, as well as the diagnostic criteria and types of otitis media classification. It is not clear if only a single specialist otologist performed the technical evaluations over the 2-week period or if more than one observer was involved. This may have resulted in interobserver bias. Intra-observer bias may also have been possible given the workload of assessing 136 participants. It would have been interesting to know if this microscope allowed for digital photography to facilitate external review by an independent expert observer. It was also not clear if cerumen removal was done consistently by a single operator (the results section mentioned that cerumen was removed manually and was halted in the event of any discomfort).
- Finally, it was not clear if the technical device required calibration during the fieldwork process; usually, a device used to take repeated measures of several participants over a short span of time requires a calibration protocol to ensure consistency and accuracy.

### 6. Explain the difference between point prevalence and incidence. (2 marks)

- The two measurements can complement each other and provide a full picture but are often confused. The incidence is a measure of the rate at which new cases of disease appear over a time period, whereas the prevalence is the total number of cases of a disease at or during a specific point in time. It is often referred to as a ‘photograph or snapshot’ of a point in time (point prevalence).
- Prevalence describes the proportion of the population with a specific characteristic, regardless of when they first developed the characteristic. This means that prevalence includes both new and pre-existing cases, whereas incidence is limited to new cases only.

### 7. Critically appraise the analysis and conclusions of the study. (4 marks)

- The authors calculated the prevalence of otitis media appropriately and used well-defined otomicroscopic definitions for the different diagnoses. However, they proceeded to compare prevalence rates between two different age groups using Pearson’s χ2 (chi-squared) test but did not indicate that this comparison will be done in their original objectives. They also did not indicate that their sample size calculation anticipated an analytical component to their study and not just a descriptive point prevalence. The authors did find a statistically significant finding during this comparative analysis that otomicroscopy-confirmed otitis media was more prevalent in the younger group of participants (preschool) compared with the older group of participants (school-going age).
- The subtypes of diagnosed otitis media confirmed that otitis media with effusion (OME) was more frequently diagnosed in the younger group, while the most severe form of otitis media, chronic suppurative otitis media (CSOM), was more common in the older group. The prevalence of CSOM for the total study sample was 6.6%, which constitutes a high-risk population. The CSOM prevalence in the older group was even higher at 9.3%, which is rated as the highest prevalence based on the World Health Organization (WHO) classification system cited by the authors.
- The authors admitted to several study design limitations, including the sample size and the lack of information on comorbid medical conditions such as HIV and TB status, as well as host-related and environmental factors, including nutritional status.
- Although the authors concur that the HIV prevalence of the population could likely contribute to the higher prevalence of otitis media, they still problematically proceed to engage with the findings as if they represent the larger population of children in primary health care settings. This is most starkly noted in their conclusion, where the HIV positivity of the children in the study is omitted.

### 8. Use a structured approach (e.g. relevance, education, applicability, discrimination, evaluation, reaction [READER]) to discuss the value of these findings to your practice (6 marks)

The READER format may be used to answer this question:

- Relevance – Is it relevant to family medicine and primary care?
- Education – Does it challenge existing knowledge or thinking?
- Applicability – Are the results applicable to my practice?
- Discrimination – Is the study scientifically valid enough?
- Evaluation – Given the above, how would I score or evaluate the usefulness of this study to my practice?
- Reaction – What will I do with the study findings?

*The answer may be a subjective response but should be one that demonstrates a reflection on the possible changes within the student’s practice within the South African public health care system. It is acceptable for the student to suggest how their practice might change within other scenarios after graduation (e.g. private general practice). The reflection on whether all important outcomes were considered is therefore dependent on the reader’s perspective (is there other information you would have liked to see?).*

*A model answer could be written from the perspective of the family physician employed in the South African district health system:*

- R: This study is relevant to the African primary care context, as children presenting to PHC facilities with otitis media are a common phenomenon, and there is a need to diagnose complicated otitis media such as OME and CSOM early to prevent complications.
- E: The authors made the case that this is the first otitis media prevalence study in a PHC setting in South Africa, especially given their use of the enhanced diagnostic instrument, the otomicroscope operated by a specialist otologist. The study’s novelty is limited by several design flaws, however.
- A: It is not possible to generalise the study findings to the wider South African setting, as the study was conducted in a specialist HIV and TB PHC facility using a small sample with a nonprobability sampling method (consecutive sampling).
- D: In terms of discrimination, the concern lies in the study design as mentioned above (small sample and sampling method). The diagnostic accuracy is noted as the authors employed a superior diagnostic technique with clearly focused and defined diagnostic criteria. The data collection process and risk for bias are not adequately presented in the methods section. Using a reporting guideline such as the Strengthening the Reporting of Observational Studies in Epidemiology (STROBE) checklist for observational studies would have enabled the reader to make a better judgement in terms of assessment of internal validity.
- E: The study findings may be relevant to consider when planning coordination of care for children in a similar PHC facility. It is important to consider the presence of complicated otitis media in children, especially those with comorbid conditions. It is also important to note the low incidence of reported symptoms in the 2 weeks prior to otomicroscopy. However, given the concerns described above regarding the study design and reporting, the findings are not generalisable to the typical South African PHC facility setting.
- R: The study findings are limited by the study setting and design flaws. However, this does not detract from the need to ensure appropriate care for children at risk for complicated otitis media. This would include increasing and augmenting routine screening services with specialised otomicroscopy services where feasible. More research in typical PHC settings with larger samples and more comprehensive data collection tools is warranted to strengthen the case made by the authors.

**Further reading**

- Pather M. Evidence-based Family Medicine. In: Mash B, editor. Handbook of Family Medicine. 4th ed. Cape Town: Oxford University Press, 2017; 430–453.
- Riegelman RK. Studying a Study and testing a test. How to read the medical evidence. 5th ed. Lippincott Williams & Wilkins; 2005.
- MacAuley D. READER: An acronym to aid critical reading by general practitioners. BR J Gen Pract. 1994;44(379):83–5.
- Von Elm E, Altman DG, Egger M, Pocock SJ, Gøtzsche PC, Vandenbroucke JP, Strobe Initiative. The Strengthening the Reporting of Observational Studies in Epidemiology (STROBE) statement: guidelines for reporting observational studies. Ann. Intern. Med. 2007;147(8):573– 577. [cited 2022 Sept 19]. Available from: https://www.equator-network.org/reporting-guidelines/strobe/
- The Critical Appraisals Skills Programme (CASP). CASP checklists. [online] 2022. [cited 2022 Sept 19]. Available from: https://casp-uk.net/casp-tools-checklists/

## Objectively structured clinical examination (OSCE) station scenario

**Objective**

This station tests the candidate’s ability to manage a patient with persistent dizziness.

**Type of station**

Integrated consultation.

**Role player**

Simulated patient: male or female adult.

**Instructions to the candidate**

- You are the family physician working at a community health centre. The medical officer asked you to see a patient with persistent dizziness, who presented to the emergency room.
- Your task: please consult with this patient and develop a comprehensive management plan.
- You do not need to examine this patient. All examination findings will be provided on request.

## Instructions to the examiner

**FIGURE 1:**
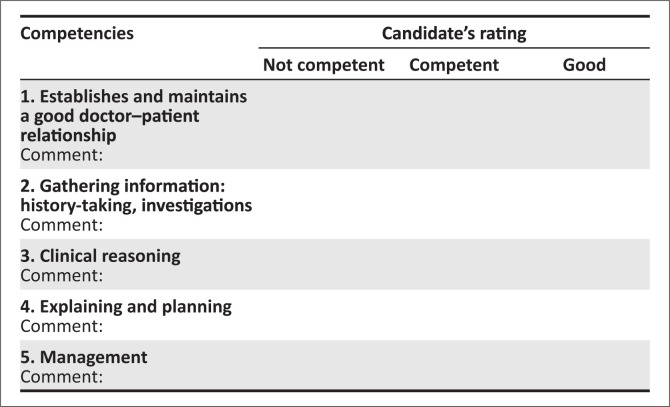
Marking sheet for objectively structured clinical examination station scenario.

- This is an integrated consultation station in which the candidate has 15 minutes.
- Familiarise yourself with the assessor guidelines that detail the required responses expected from the candidate.
- No marks are allocated. In the marks sheet (Figure 1), tick off one of the three responses for each of the competencies listed. Make sure you are clear on what the criteria are for judging a candidate’s competence in each area.
- Provide the following information to the candidate when requested: examination findings.
- Please switch off your cell phone.
- Please do not prompt the student.
- Please ensure that the station remains tidy and is reset between candidates.

**Guidelines to examiner**

The aim is to establish that the candidate can diagnose vertigo, identify possible causes (cerebellar stroke with underlying hypercholesterolaemia) and develop an effective and safe management plan.

**Working definition of competent performance:** the candidate *effectively completes the task* within the allotted time, in a manner that *maintains patient safety,* even though the execution may not be efficient and well structured.

- *Not competent:* patient safety is compromised (including ethical-legally) or task is not completed.
- *Competent:* the task is completed safely and effectively.
- *Good:* in addition to displaying competence, the task is completed efficiently and in an empathic, patient-centred manner (acknowledges patient’s ideas, beliefs, expectations, concerns or fears).

- **Establishes and maintains a good clinician–patient relationship**
  The *competent candidate* is respectful, engaging with the patient in a dignified manner. The *good candidate* is empathic, compassionate and collaborative, facilitating patient participation in key areas of the consultation.
- **Gathering information**
  The *competent candidate* gathers sufficient information to establish a diagnosis (*acute vertigo, asks questions aimed at localising the problem and enquires about some psychosocial related to the problem*)
  The *good candidate* additionally has a structured and holistic approach (*enquiring about the causes of vertigo and assessing the impact on the emotional, social and occupational aspects of the patient’s life*).
- **Clinical reasoning**
  The *competent candidate* identifies the diagnosis (*acute vertigo due to a central cause, impacting the patient’s work performance as a bus driver*).
  The *good candidate* makes a specific diagnosis (*acute vertigo, likely due to a cerebellar stroke, with underlying possible familial hypercholesterolaemia, with major long-term occupational implications*).
- **Explaining and planning**
  The *competent candidate* uses clear language to explain the problem to the patient and uses strategies to ensure patient understanding (*questions OR feedback OR reverse summarising*).
  The *good candidate* additionally ensures that the patient is actively involved in decision-making, paying particular attention to knowledge-sharing and empowerment.
- **Management**
  The *competent candidate* makes arrangements for urgent referral to a specialist physician or neurologist for further investigations (*computerised tomography* [CT] *scan or magnetic resonance imaging* [MRI]) as an inpatient.
  The *good candidate* additionally addresses psychosocial issues comprehensively and may start the process of a follow-up plan being in place when the patient returns from the hospital.

**Examination findings**

- Body mass index – 24 kg/m^2^
- Blood pressure – 138/94 mmHg, heart rate: 104 beats/min
- Haemoglobin – 13.5 gm/dL
- Random blood glucose (HGT) – 5.9 mmol/L
- Urinalysis – No abnormalities
- Ears – Normal hearing bilaterally; no abnormalities on visual inspection, including otoscope; Dix-Hallpike manoeuvre negative.
- Eyes – Xanthelasma on both eyelids; nystagmus on lateral gaze; normal vision, specifically no diplopia.
- Cardio-respiratory systems – No abnormalities.
- Abdomen – No abnormalities.
- Neuro – Marked ataxic gait; fine tremor at rest: unable to write own name; power, reflexes and sensation intact in all limbs.

## Role player instructions

- **Appearance and behaviour**

Male or female adult, calm, 40–50 years old.

- **Opening statement**

‘Hello, Doctor. I’m having this dizziness all the time, since yesterday, and feeling nauseous.’

**History**

- Open responses: Freely tell the doctor
  ■ You were feeling very well yesterday morning. Around lunchtime, you suddenly started getting dizzy and vomited twice. You had to leave work, then slept at home until this morning, but it is not better.
- Closed responses: Only tell the doctor if asked
  ■ It feels like the room is spinning around you. Makes it difficult to walk. Not worsened by any specific positions.
  ■ Nauseous all the time, especially when you are moving.
  ■ You have no funny ringing noises or deafness in any of your ears.
- Your medical history
  ■ Diagnosed with high cholesterol at the age of 34 years. Did not want to use medication – just eating healthily and exercising occasionally. Cholesterol is a family problem; your brother and mother also have cholesterol problems, but you are unsure if they take medication.
  ■ You do not smoke, drink alcohol very little and exercise by walking once a week.
- Ideas, concerns and expectations
  ■ Your major concern is to get rid of this dizziness.
  ■ It affects your work as a bus driver.

**Further reading**

- Department of Health. Acute Vertigo. In: Standard Treatment Guidelines, Adult Hospital level. Pretoria: Department of Health; 2019.

